# Chinese population may require further venetoclax dose reduction beyond guidelines when combined with voriconazole: real-world evidence from China

**DOI:** 10.3389/fphar.2025.1539233

**Published:** 2025-05-30

**Authors:** Rongrong Wang, Yanfen Li, Ran Zhang, Yuan Ren, Yifan Liu, Tianlin Wang, Jin Wu, Xiangjin Zheng, Shu Li, Yu Jing

**Affiliations:** ^1^ Department of Pharmacy, Medical Supplies Center, Chinese PLA General Hospital, Beijing, China; ^2^ Department of Hematology in the Fifth Medical Center of PLA General Hospital, Beijing, China

**Keywords:** venetoclax, azoles, drug-drug interactions, safety, economics

## Abstract

**Objective:**

Azole antifungals inhibit the enzyme cytochrome P450 3A4 (CYP 3A4), increasing venetoclax (VEN) levels and the risk of serious adverse reactions. Dose adjustments for VEN with voriconazole (VOR) vary in studies. The drug-drug interactions (DDI), safety and economic implications of VEN with VOR in the Chinese population, who may have unique drug exposures, are unclear. This study aims to address these uncertainties and provide guidance for clinical practice.

**Methods:**

The DDI were assessed by measuring trough (Ctrough) and peak (Cpeak) levels of VEN and concomitant azoles after ≥7 days of continuous administration. Safety and economic implications were evaluated based on the duration of cytopenias and hospitalization costs for Chinese patients with acute myeloid leukemia (AML) across three groups: VEN 400 mg, VEN 100 mg combined with posaconazole (POS) (VEN 100 mg + POS) and VEN 100 mg combined with VOR (VEN 100 mg + VOR).

**Results:**

VOR was able to significantly increase the Ctrough (3.40 vs. 0.99, *p < 0.05*) μg/mL and Cpeak (3.71 vs. 2.22, *p < 0.05*) μg/mL of VEN 100 mg compared to VEN 400 mg alone. This increase in the plasma concentration of VEN may result in a longer duration of days to white blood cell (WBC) > 2000 cells/mm^3^ (25 vs. 13, *p < 0.05*) and a higher likelihood of increased hospitalization costs (140,469 vs. 73,513, *p = 0.068*) compared to VEN 400 mg alone.

**Conclusion:**

Chinese population may require further dose reduction of VEN beyond guideline recommendations when combined with VOR.

## Introduction

VEN is the first and currently the only B-cell lymphoma-2 (BCL-2) inhibitor included in the guidelines. It can significantly improve survival in patients with AML and chronic lymphocytic leukaemia (DiNardo et al., 2020; Lachowiez et al., 2020; Lasica and Anderson, 2021). However, chemotherapy for haematological malignancies often results in prolonged and severe neutropenia, which can lead to invasive fungal infections. The mortality rate of patients diagnosed with invasive fungal infections is as high as 11.7% (Sun et al., 2015). Therefore, antifungal prophylaxis and treatment are required during treatment (Rausch et al., 2021). POS and VOR are first-line therapies for the prophylaxis and treatment of fungal infections in hematologic diseases (Chinese Association Hematologists, Chinese Invasive Fungal Infection Working Group, 2020). These azole antifungals are frequently co-administered with VEN in clinical practice. VEN is mainly metabolised by CYP 3A4 and both POS and VOR are strong inhibitors of the same enzyme. Therefore, it is important to note that there is a potential for DDI to increase plasma concentration of VEN (Agarwal et al., 2017b; Bhatnagar et al., 2021). This increased concentration may lead to safety concerns such as febrile neutropenia, pneumonia and sepsis (DiNardo et al., 2018). So, it is crucial to consider dose adjustment for VEN in clinical practice.

Studies have shown that the combination of 100 mg VEN and POS increased the mean maximum concentration (Cmax) and area under the concentration-time curve from 0 to 24 h (AUC_0-24_) of VEN by 93% and 155%, respectively, compared to 400 mg VEN alone (Agarwal et al., 2017a; Venclexta, 2018 (venetoclax) [package insert]. Chicago, 2018). As a result, guidelines suggest reducing the dose of VEN to 70 mg when co-administered with POS, and adjusting the dose to 100 mg with other strong CYP 3 A inhibitors (Oncology, 2021). However, recommendations for dose adjustment of VEN in combination with VOR are not consistent across studies (Izutsu et al., 2021; Dong et al., 2022; Kobayashi et al., 2022). VEN levels may also vary between Chinese and non-Asian populations (Cheung et al., 2018). Therefore, further evaluation is needed to determine the appropriateness of reducing VEN to 100 mg when used with VOR.

The objectives of this study were to: (1) provide quantitative insights into the risk of DDI between VEN and VOR; (2) compare data on plasma concentrations, duration of cytopenias and hospitalization costs to inform potential dose adjustments.

## Material and methods

### Patients and study design

This study aimed to explore the DDI, safety and economics of VEN with VOR in the Chinese patients with AML, utilizing the DCAG/CACAG regimen. Patients were treated at our hospital between 2022 and 2023.

**FIGURE 1:**
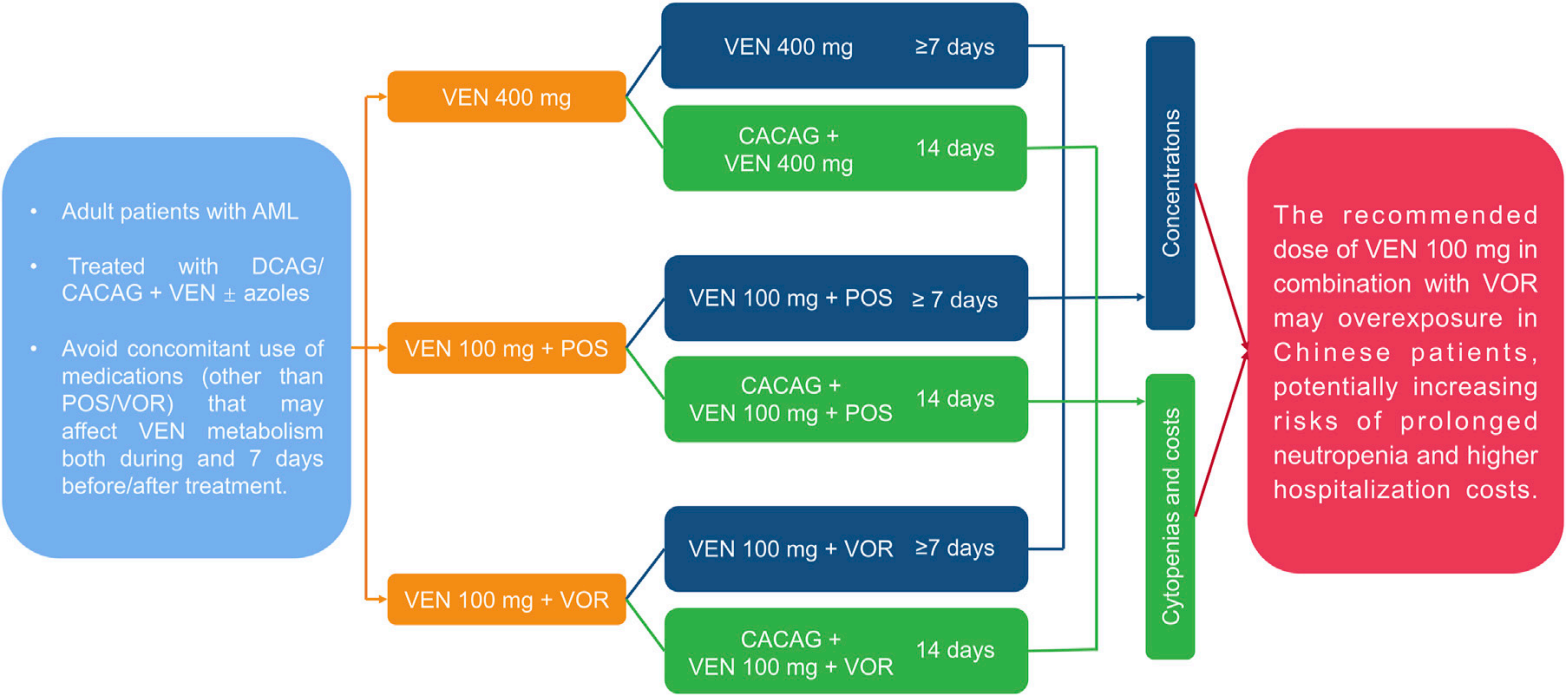
A comprehensive methodological framework summarizing the structure and steps of scientific research. AML: acute myeloid leukemia; VEN: venetoclax; POS: posaconazole; VOR: voriconazole; DCAG/CACAG: chemotherapy regimen.

A comprehensive methodological framework is illustrated in Figure 1, summarizing the research structure and steps. This retrospective analysis included adult AML patients (≥18 years) who received DCAG/CACAG + VEN alone or in combination with VOR or POS. The inclusion criteria were: Eastern Cooperative Oncology Group (ECOG) score ≤3; age 18–70; no severe allergies or psychiatric disorders; liver function within 2.5 times the upper limit of normal; bilirubin ≤2.0 times the upper limit; creatinine within normal limits; no significant cardiac dysfunction; and no uncontrolled active infections. Exclusion criteria included: concomitant use of medications (other than POS/VOR) that may affect VEN metabolism both during and 7 days before/after treatment; and unsuitability for the study such as recent surgery. CACAG: cytarabine 75–100 mg/m^2^ q12 h d1-d5; azacitidine 50–75 mg/m^2^/d1-d7; chidamide 30 mg/d1 d4; aclarubicin 20 mg/d1 d3 d5; granulocyte colony-stimulating factor (G-CSF) 300 μg/d until WBC count reached 2000 cells/mm^3^. DCAG: decitabine 20 mg/m^2^/d, d1-5; cytarabine 50–100 mg/m^2^/d, d1-5; aclarubicin 20 mg/d1-5; G-CSF 300ug/d or PEGylated recombinant human granulocyte colony-stimulating Factor (PEG-CSF) 6 mg once a week until WBC count reached 2000 cells/mm^3^. Patients were divided into three groups: VEN 400 mg, VEN 100 mg + POS (oral suspension, 200 mg, tid), and VEN 100 mg + VOR (tablet/injection 200 mg bid). Data were obtained from the medical records.

To assess the impact of azoles coadministration on VEN pharmacokinetics, steady-state Ctrough and Cpeak of VEN were collected following ≥7 days of treatment across the three groups. All Ctrough were collected within 30 min before drug administration. Cpeak for VEN, POS, VOR tablet, and VOR injection were sampled approximately 7 h, 3 h, 1 h and immediately (0 h) after administration, respectively.

To minimize variability from chemotherapy regimens, we restricted our analysis to CACAG patients when assessing cytopenias and hospitalization costs. Details of medication use met the following criteria: ① VEN 400 mg: CACAG + VEN 100 mg d1, 200 mg d2, and 400 mg d3-d14, ② VEN 100 mg + POS: CACAG + POS with VEN 100 mg qd for 14 days, ③ VEN 100 mg + VOR: CACAG + VOR with VEN 100 mg qd for 14 days. The duration of neutropenia (WBC <1,000/2,000 cells/mm^3^) and thrombocytopenia (PLT <50,000/100,000 cells/mm^3^) was calculated. Time to neutrophil recovery was calculated from the date of WBC <1,000/2000 cells/mm^3^ to the two consecutive or first measurements of WBC >1,000/2000 cells/mm^3^ or WBC >2000/4,000 cells/mm^3^. Similarly, PLT <50,000/100,000 cells/mm^3^ was also counted. If the patient’s WBC/PLT did not recover to the statistical endpoint, the discharge time would be used. There was partial patient overlap between the cohort with plasma concentration data and the cohort analyzed for cytopenia/hospitalization costs.

Meanwhile, the hospitalization costs of each chemotherapy session for these patients were collected. The hospitalization costs in our study were collected as the sum of all fees for healthcare services incurred during a 1-cycle hospitalization. Services included basic and special inpatient care, initial consultation and examination, imaging, pharmacy, injections, treatments, invasive procedures and pre-discharge counselling; fees were calculated in China Yuan (CNY).

### Follow-up and bioanalytical methods

Patients were evaluated for 1-cycle DDI, safety, and economic outcomes. DDI assessments involved quantification of VEN, POS, and VOR plasma concentrations using a validated LC-MS/MS method (Mei et al., 2020; Lei et al., 2021), with POS detection following the same protocol as VOR. Safety analyses focused on cytopenia incidence as determined by laboratory testing. Economic evaluations encompassed total hospitalization costs, with patient data extracted from inpatient records. The study follow-up period extended through December 2023, with endpoints defined as either reaching the cutoff date or hospital discharge.

### Statistical analyses

Statistical analysis was performed using GraphPad Prism version 9, employing non-parametric tests due to the small sample size. Data are presented as medians (Q25, Q75). Comparisons across groups were made using Kruskal–Wallis test. The Kaplan-Meier method was used to estimate distributions of and median times to ANC and PLT recovery. Log rank test was used to compare these time-to-event outcomes among different subgroups. Correlation analyses used Pearson’s or Spearman’s tests, with *p < 0.05* indicating significance.

## Results

### Patient characteristics

Table 1 shows the characteristics of the patients included in the study. In the plasma concentrations study. 17 patients received VEN 400 mg, 10 patients received VEN 100 mg + POS, and 12 patients received VEN 100 mg + VOR. VOR consists of 7 injections and 5 tablets, while POS is only available as an oral suspension.

**TABLE 1:** Patients and treatment characteristics.

Characteristics	VEN 400 mg	VEN 100 mg + POS	VEN 100 mg + VOR
Plasma concentrations study (DCAG/CACAG)			
Sex	Female 9; Male 8	Female 6; Male 4	Female 7; Male 5
Age (year)	57 (43–59)	59 (43–64)	59 (51–60)
Weight (kg)	65 (61–72)	64 (58–72)	64 (62–68)
BMI (kg/m^2^)	23.8 (22.8–25.3)	22.9 (22.5–25.9)	23.7 (22.8–24.4)
Azoles	-	Oral suspension 10	Injection 7; Tablet 5
Cytopenias and costs study (CACAG)			
Sex	Female 4; Male 1	Female 3; Male 2	Female 1; Male 4
Age (year)	30 (28–51)	42 (39–43)	55 (43–62)
Weight (kg)	63 (61–65)	56 (51–71)	62 (60–63)
BMI (kg/m^2^)	23.5 (21.9–23.6)	21.3 (20.3–22.8)	22.7 (22.5–24.9)
*De novo* AML	No prior therapy 2 Prior therapy 3	No prior therapy 2 Prior therapy 3	No prior therapy 2 Prior therapy 3
Azoles	-	Oral suspension 5	Injection 2; Tablet 3

Data expressed as median (range). POS, posaconazole; VOR, voriconazole; VEN, venetoclax; BMI, body mass index; AML, acute myeloid leukemia; DCAG/CACAG, chemotherapy regimen.

In the cytopenias and hospitalization costs study, there were five patients in each of the three groups: VEN 400 mg, VEN 100 mg + POS and VEN 100 mg + VOR. Of these patients, two out of five in each group had received no prior therapy, while the remaining three patients had received prior therapy.

### The Ctrough and Cpeak of VEN in VEN 100 mg + VOR are significantly higher than in VEN 400 mg alone or VEN 100 mg + POS

**FIGURE 2:**
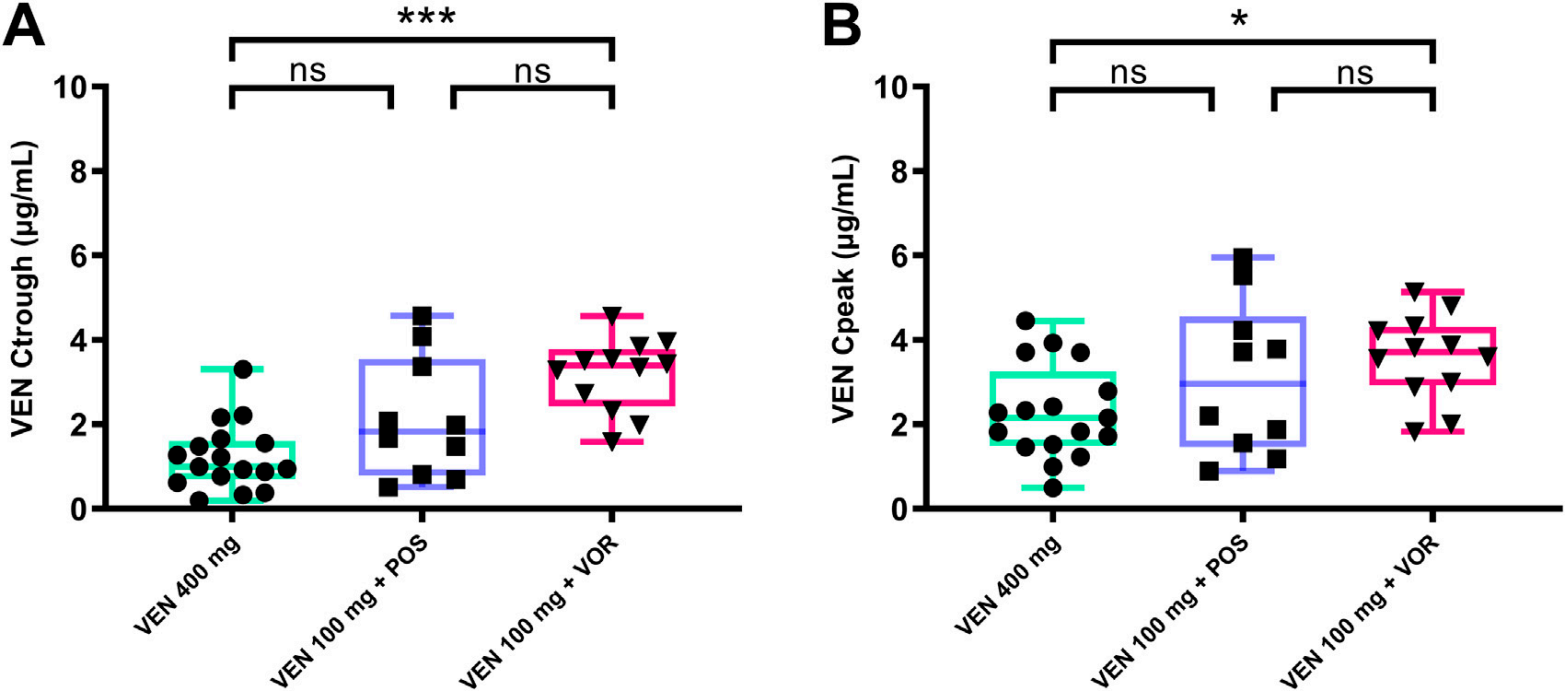
VEN steady-state Ctrough **(A)** and Cpeak **(B)** by groups: VEN 400 mg, VEN 100 mg + POS, VEN 100 mg + VOR. *p < 0.05 **p < 0.01 ***p < 0.001 versus control. Ctrough: trough concentrations; Cpeak: peak concentrations; POS: posaconazole; VOR: voriconazole; VEN: venetoclax.

As shown in Figure 2, the concentration of VEN 400 mg in our study is similar to the Ctrough (0.99 vs. 1.00) μg/mL and Cpeak (2.22 vs. 2.30) μg/mL in previous studies (Agarwal et al., 2017a). The Ctrough and Cpeak of VEN increased progressively in the VEN 400 mg, VEN 100 mg + POS, and VEN 100 mg + VOR. VOR was able to significantly increase the Ctrough (3.40 vs. 0.99, *p < 0.05*) μg/mL (Figure 2A) and Cpeak (3.71 vs. 2.22, *p < 0.05*) μg/mL (Figure 2B) of VEN (100 mg), with the Ctrough of VEN (100 mg) being substantially higher than that of VEN 400 mg (Figure 2A). No statistically significant differences were observed between the VEN 100 mg + POS group versus either VEN 400 mg or VEN 100 mg + VOR.

### Higher therapeutic target achievement rates with VOR compared to POS

**FIGURE 3:**
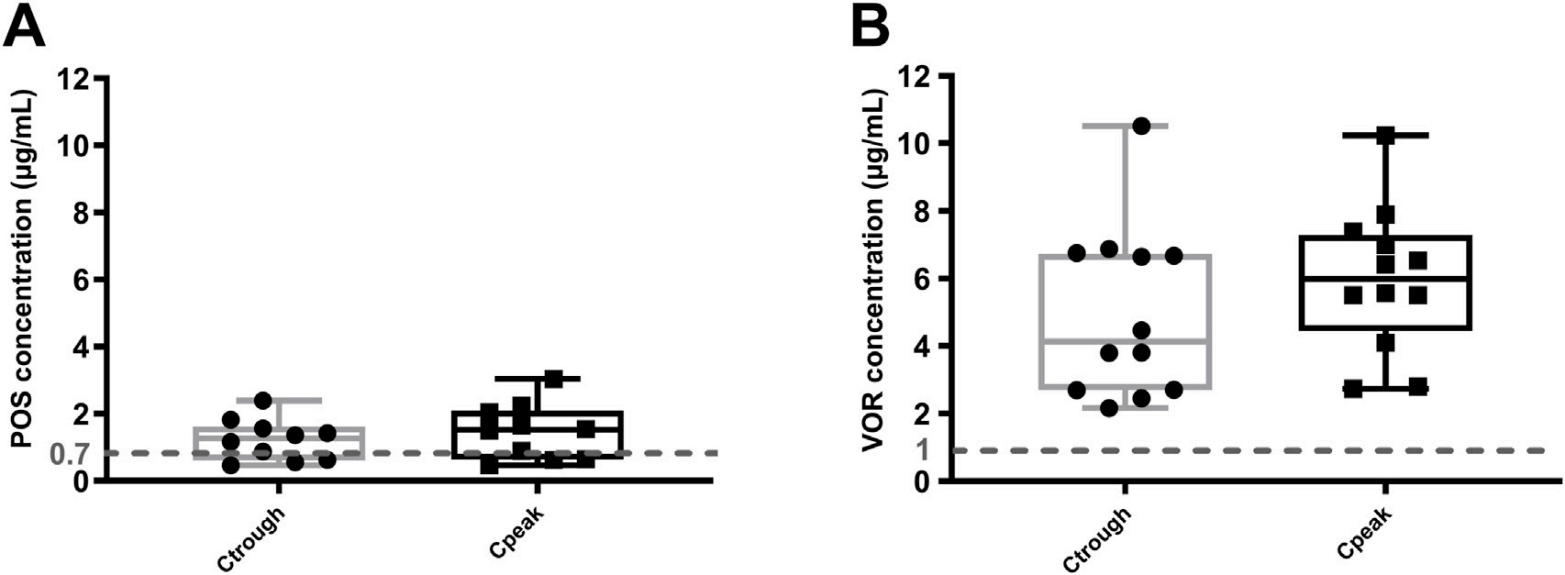
Steady-state Ctrough and Cpeak of POS **(A)** and VOR **(B)**. Dashed line: azoles minimum target concentration. Ctrough: trough concentrations; Cpeak: peak concentrations; POS: posaconazole; VOR: voriconazole.

VOR concentrations were all above the recommended lower limit of 1 μg/mL (Pascual et al., 2008), as shown in Figure 3. However, 70.0% (7/10) of patients taking the POS oral suspension had concentrations exceeding the recommended lower limit by 0.7 μg/mL (Jang et al., 2010). The median Ctrough and Cpeak of POS were 1.27 (0.68, 1.53) μg/mL and 1.52 (0.70, 1.95) μg/mL (Figure 3A), respectively. The median Ctrough and Cpeak of the VOR were 4.13 (2.70, 6.69) μg/mL and 5.99 (4.15, 7.09) μg/mL (Figure 3B), respectively. Additionally, there is a strong correlation between VEN Ctrough and POS Ctrough (r = 0.850, *p = 0.002*), but no correlation with VOR Ctrough (r = 0.004, *p = 0.989*).

### The VEN 100 mg + VOR had prolonged cytopenias and higher bacterial infection rates than VEN 400 mg or VEN 100 mg + POS

**FIGURE 4:**
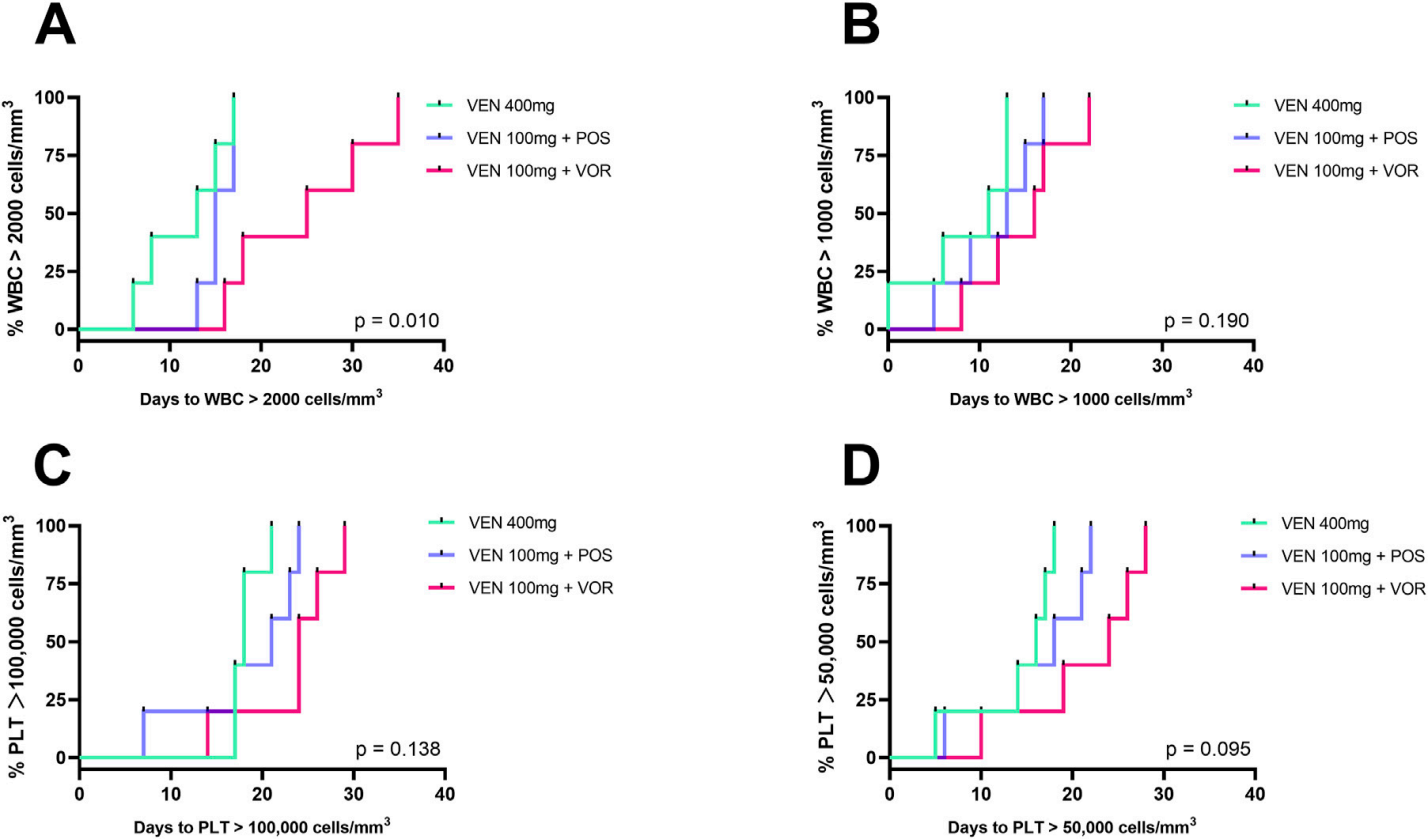
Duration of cytopenias in CACAG-treated Chinese patients by groups: VEN 400 mg, VEN 100 mg + POS, VEN 100 mg + VOR. **(A)**: Days to WBC >2000 cells/mm^3^; **(B)**: Days to WBC >1,000 cells/mm^3^; **(C)**: Days to PLT >100,000 cells/mm^3^; **(D)**: Days to PLT >50,000 cells/mm^3^; Key **(A)**: Only VEN 100 mg + VOR showed significantly prolonged recovery (WBC >2,000/mm^3^) vs. VEN 400 mg (*p < 0.05*). POS: posaconazole; VOR: voriconazole; VEN: venetoclax; PLT: thrombocytopenia; WBC: white blood cell.

As shown in Figure 4, days to PLT >50,000/100,000 cells/mm^3^ and days to WBC> 1,000/2000 cells/mm^3^ increased progressively in the VEN 400 mg, VEN 100 mg + POS, and VEN 100 mg + VOR. Notably, only the VEN 100 mg + VOR group had a significantly longer duration of days to WBC >2000 cells/mm^3^ than VEN 400 mg group (25 vs. 13, *p < 0.05*). It is worth noting that the duration of granulocyte support was the same across groups, but there was a difference in platelet support. Consistent with the results in Figure 2, there was no statistical difference between the VEN 100 mg + POS and either the VEN 400 mg or the VEN 100 mg + VOR groups. Bacterial infection rates differed across groups: 60% (3/5) in VEN 100 mg + VOR, 40% (2/5) in VEN 100 mg + POS, and 0% (0/5) in VEN 400 mg. All patients completed therapy, and no mortality events occurred.

### Hospitalization costs for VEN 100 mg + VOR are higher than for VEN 400 mg alone or VEN 100 mg + POS

**FIGURE 5:**
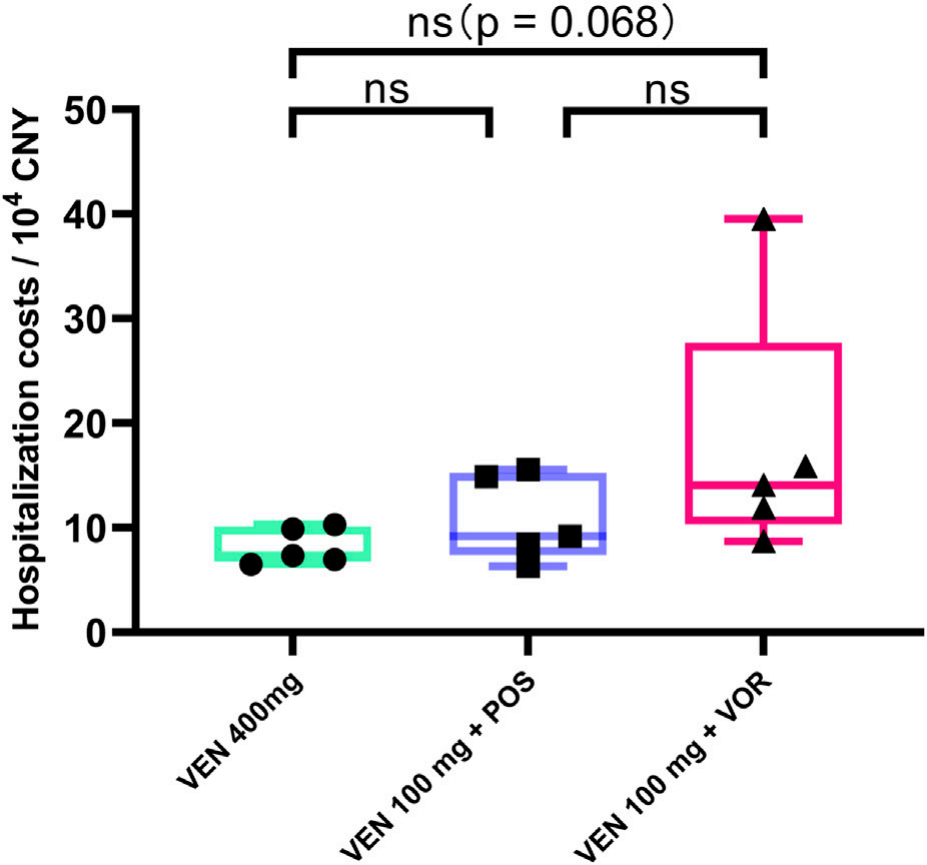
Hospitalization costs in CACAG-treated Chinese patients by groups: VEN 400 mg, VEN 100 mg + POS, VEN 100 mg + VOR. POS: posaconazole; VOR: voriconazole; VEN: venetoclax.

Hospitalization costs exhibit an increasing trend in the VEN 400 mg, VEN 100 mg + POS, and VEN 100 mg + VOR (73,513 vs. 91,667 vs. 140,469, *p = 0.068*). However, these intergroup differences did not reach statistical significance (Figure 5). A comprehensive comparison of plasma VEN concentrations, duration of cytopenias, and hospitalization costs among the three treatment regimens is presented in Table 2.

**TABLE 2:** Comparison of plasma concentrations of VEN, the duration of cytopenias, and hospitalization costs among different treatment groups: VEN 400 mg, VEN 100 mg + POS, and VEN 100 mg + VOR.

VEN exposure & outcomes	VEN 400 mg	VEN 100 mg + POS	VEN 100 mg + VOR
Ctrough of VEN	0.99 (0.33–3.30)	1.83 (0.52–4.57)	3.4 (1.59–4.56) ***
Cpeak of VEN	2.22 (0.49–4.45)	2.96 (0.89–5.59)	3.71 (1.83–5.14) *
Days to WBC >2000 cells/mm^3^	13 (6–17)	15 (13–17)	25 (16–35) **
Days to WBC >1,000 cells/mm^3^	11 (0–13)	13 (5–17)	16 (8–22)
Days to PLT >100,000 cells/mm^3^	18 (17–21)	21 (7–24)	24 (14–29)
Days to PLT >50,000 cells/mm^3^	16 (5–18)	18 (6–22)	24 (10–28)
Bacterial infection rates	0% (0/5)	40% (2/5)	60% (3/5)
Hospitalization costs	73,513 (65,392–103,089)	91,667 (63,116–103,089)	140,469 (87,017–395,048)

Data expressed as median (range) or n (%); *p < 0.05 **p < 0.01 ***p < 0.001 versus VEN, 400 mg. VEN, venetoclax; POS, posaconazole; VOR, voriconazole; PLT, thrombocytopenia; WBC, white blood cell; Ctrough, trough concentrations; Cpeak, peak concentrations.

## Discussion

This report represents the first real-world investigation of the effects of VOR on VEN plasma concentrations, duration of cytopenias, and hospitalization costs in Chinese individuals. Our preliminary findings suggest that the guideline-recommended dosage may not be suitable for Chinese patients and that further dose reductions might be necessary.

Our study revealed that both the Ctrough and Cpeak of VEN 100 mg + VOR were significantly higher compared to VEN 400 mg alone. Presently, real-world data on VEN concentrations when co-administered with VOR are sparse. A previous study involving four patients found that co-treatment with VOR significantly increased the Ctrough of VEN (100 mg) (Gao et al., 2023), aligning closely with our results (3.0 vs. 3.4). Another study used population pharmacokinetic modeling to analyze the DDI between VEN and VOR, suggesting a 100 mg dose of VEN in the presence of VOR. However, this model was not validated using real-world DDI concentration data (Dong et al., 2022). The rationale for considering 100 mg VEN as the appropriate dose in the study was based on the assumption that increased VEN levels correlate with VOR concentrations. In contrast, our study found a non-linear relationship between VOR and VEN concentrations, suggesting that further validation is needed to confirm whether VEN concentrations indeed positively correlate with VOR concentrations at target VOR levels.

We observed no statistically significant differences in VEN Ctrough, Cpeak, or neutropenia duration between VEN 400 mg and VEN 100 mg + POS, consistent with current guidelines recommending VEN dose reduction to 100 mg during POS coadministration. Notably, Asian populations, particularly Chinese patients, exhibit higher VEN exposure than non-Asian cohorts (Kobayashi et al., 2022; Gao et al., 2023), suggesting that even the guideline-recommended 100 mg dose of VEN—whether combined with POS or VOR—might exceed therapeutic requirements in this population. Importantly, our study used POS oral suspension, which has substantially lower bioavailability than the enteric-coated tablets specified in guidelines (Dekkers et al., 2016). Furthermore, we observed a low target concentration achievement rate (70.0%) for POS, concomitant with a concentration-dependent escalation in VEN exposure. This positive VEN-POS correlation likely reflects insufficient POS concentrations to saturate CYP 3A/transporter inhibition, contrasting with VOR’s 100% target attainment and saturated inhibition. The inadequate POS exposure resulted in: ① attenuated VEN-POS DDI with consequently lower VEN levels; and ② subsequently reduced VEN-associated myelosuppression. This may explain both faster cytopenia recovery in VEN 100 mg + POS and the comparable Ctrough/Cpeak between VEN 400 mg and VEN 100 mg + POS. Future studies should consider POS formulations to ensure adequate exposure.

The duration of neutropenia showed a statistically significant relationship between VEN 100 mg + VOR and VEN 400 mg. Notably, the exposure-response analysis showed that VEN 400 mg daily (in combination with a hypomethylating agent as part of the CACAG/DCAG regimens) achieves maximal clinical efficacy (Brackman et al., 2022). Therefore, while VOR co-administration increases VEN exposure, this elevated concentration is unlikely to further improve remission rates. However, the higher drug exposure may contribute to an increased risk of cytopenias. There was no significant difference in days to PLT >50,000/100,000 cells/mm^3^, but there is a significant difference in days to WBC >1,000/2000 cells/mm^3^. This discrepancy might be due to consistent granulocyte support (G-CSF) while platelet support varied (recombinant human interleukin 11; recombinant human thrombopoietin; thrombopoietin receptor agonist). The time to WBC >2000 cells/mm^3^ was also significantly different, suggesting that granulocyte support primarily reduces the duration of severe neutropenia. Exposure-response analyses have shown that Asian patients are more likely to experience treatment-emergent Grade ≥3 neutropenia, regardless of whether they received VEN plus hypomethylating agent or placebo plus azacitidine (Brackman et al., 2022). Two other studies indicate the appropriateness of a 100 mg dose of VEN by demonstrating that patients who received VEN 100 mg + VOR did not experience worse hematological outcomes compared to those who did not receive VOR (Rausch et al., 2021; Hall et al., 2023). However, a major limitation of these studies is the lack of consistency in chemotherapy regimens, granulocyte, and platelet support. Of note, although the research results showed no statistical difference, the median time to recovery of absolute neutrophil count (35 vs. 38 days) and PLT (26 vs. 32 days) was slightly longer in the VOR group (Rausch et al., 2021).

Consistent with the trends observed for plasma concentrations and cytopenias, the costs of hospitalization were slightly higher when combined with VOR compared to VEN 400 mg alone (*p = 0.068*). This observation aligns with the known concentration-dependent toxicity profile of VEN, where elevated drug levels lead to more prolonged cytopenias. The resulting prolonged cytopenias not only increase bacterial infection risks but also necessitate additional supportive care, including growth factor administration and extended hospitalization - factors that collectively contribute to higher treatment costs. Our data revealed significantly higher hospitalization costs in patients with confirmed bacterial infections (155,670 vs. 78,978, *p < 0.05*). Notably, the incidence of documented bacterial infections showed a stepwise increase across treatment groups: 0% in VEN 400 mg, 40% in VEN 100 mg + POS, and 60% in VEN 100 mg + VOR groups. This pattern suggests that differential anti-infective treatment requirements may largely account for the observed cost variations.

Our research was the first to simultaneously investigate the plasma concentrations, duration of cytopenias and hospitalization costs associated with the concomitant use of VEN and VOR in the Chinese patients in a real-world setting. While current guidelines recommend reducing the VEN dose to 100 mg with VOR, pharmacokinetic data suggest potential interethnic variability in VEN exposure. Notably, variability in VEN exposure was comparable between Chinese and non-Asian populations, with similar half-life (t_1/2_). This indicates that the observed differences in VEN exposure may primarily stem from variations in bioavailability rather than clearance. VEN is principally metabolized by CYP 3A4 and serves as a substrate for P-glycoprotein (P-gp) and breast cancer resistance protein (BCRP) transporters. Given the limited functional polymorphisms identified in CYP 3A4, we hypothesize that the observed bioavailability differences may arise from: ①variability in CYP 3A expression levels; ②differences in activity/expression of gastrointestinal transport proteins; ③variations in body surface area (Cheung et al., 2018; Kobayashi et al., 2022). The Japanese study demonstrated that VEN 50 mg + VOR already achieves the effective therapeutic concentration (VEN 400 mg) (Izutsu et al., 2021; Kobayashi et al., 2022). Therefore, we speculate that a reduction in the VEN dose from 100 mg to 50 mg may be more appropriate for Chinese patients. This adjustment may lead to decreased adverse reactions, potentially lower healthcare costs related to managing these reactions, and maintain optimal therapeutic efficacy.

Nonetheless, our study has several limitations. The most significant constraint stems from the limited cohort size and heterogeneity, particularly in cost and cytopenia duration analyses (n = 5 per group), which substantially reduces statistical power and generalizability. While this sample size is consistent with prior VEN + POS DDI study (6 receiving VEN 100 mg + POS and 5 receiving VEN 50 mg + POS) (Agarwal et al., 2017a) and we implemented rigorous protocols including standardized CACAG chemotherapy, uniform G-CSF support, and balanced enrollment (2 no prior therapy +3 prior therapy patients per group), it remains below the FDA-recommended threshold of 12–20 subjects for robust DDI study. Methodologically, the use of plasma concentrations as a surrogate for remission rates may not fully capture clinical outcomes, and the economic evaluation was limited by unadjusted cost comparisons. To address these limitations, we have initiated a prospective validation study with enhanced statistical power to confirm these DDI outcomes in Chinese population.

## Conclusions

The recommended dose of VEN 100 mg in combination with VOR may lead to excessive exposure in the Chinese population, thus increasing the risk of prolonged neutropenia and the possibility of increased associated hospitalization costs. This is a clinical concern and a lower dose than 100 mg in combination with VOR may be a more appropriate option in the Chinese population.

## Data Availability

The original contributions presented in the study are included in the article/supplementary material, further inquiries can be directed to the corresponding author/s.
